# Cartilaginous Choristoma of the Gingiva: A Rare Clinical Entity

**DOI:** 10.1155/2014/246965

**Published:** 2014-12-18

**Authors:** Ramalingam Suganya, Narasimhan Malathi, Subramani Vijaya Nirmala, Chinnaswami Ravindran, Harikrishnan Thamizhchelvan

**Affiliations:** ^1^Department of Oral Pathology & Microbiology, Faculty of Dental Sciences, Sri Ramachandra University, No. 1 Ramachandra Nagar, Porur, Chennai, Tamil Nadu 600116, India; ^2^Department of Oral & Maxillofacial Surgery, Faculty of Dental Sciences, Sri Ramachandra University, No. 1 Ramachandra Nagar, Porur, Chennai, Tamil Nadu 600116, India

## Abstract

Choristomas are rare entities which are aggregates of microscopically normal cells or tissues in aberrant locations. They are a “heterotopic” rest of cells, as they appear as a tumor-like mass. Herein we report a case of cartilaginous choristoma in a 54-year-old male who presented with a swelling on right lower gingiva. The histopathological examination revealed features of a well circumscribed mass of mature cartilage in a dense fibrous connective tissue stroma.

## 1. Introduction

Choristoma, a congenital anomaly, is better described as a “heterotopic” rest of cells [1]. Choristoma is a more acknowledged term and it was first described by Krolls et al. as tumor-like growths of microscopically common tissue in an unusual location [2]. In the oral cavity, it is considered as tumor-like growth that has developed from collection of primordial cells placed at a position far-off from the original tissue or organ. It may consist of large number of cartilages, bones, fat cells, neural elements, glial tissues, respiratory tissues, thyroid glands, and intestinal mucosa [3].

Cartilaginous choristomas are rare entities in the oral cavity region which are composed of mature hyaline cartilage. This paper reports one such rare case of cartilaginous choristoma of the gingiva.

## 2. Case Report

**Figure 1:**
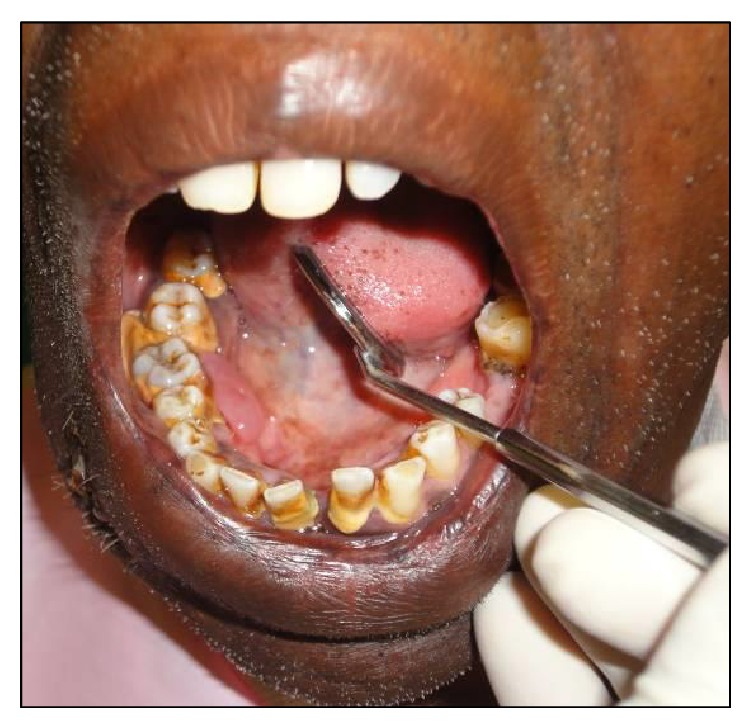
Clinical photograph of pre-op intraoral choristoma.

**Figure 2:**
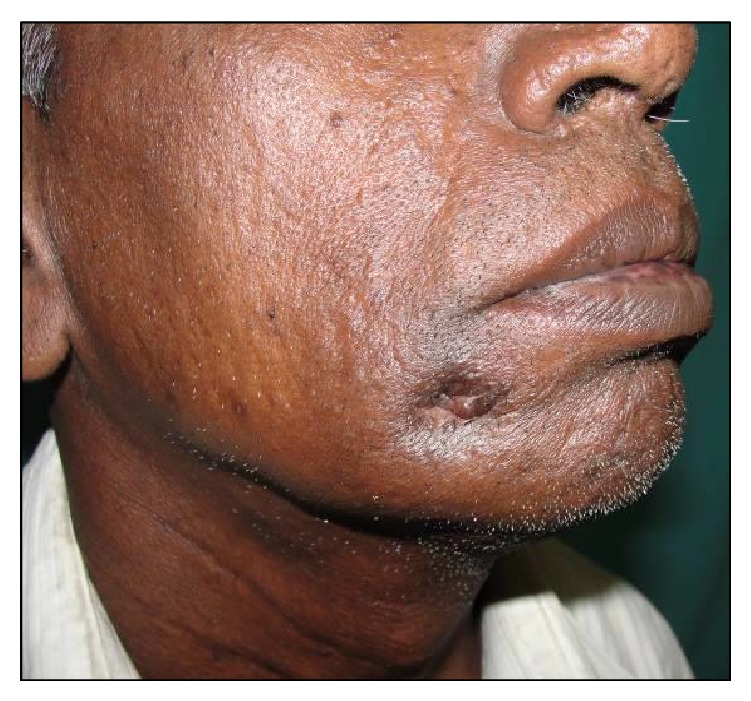
Sinus opening on right side of the face.

**Figure 3:**
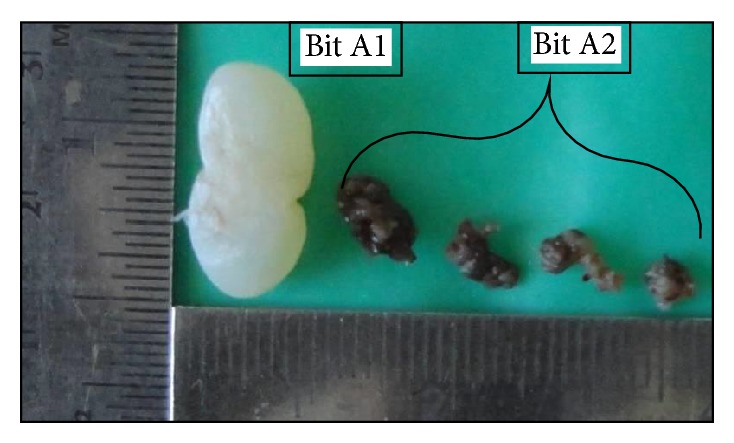
Gross specimen photograph of the incisional biopsy.

**Figure 4:**
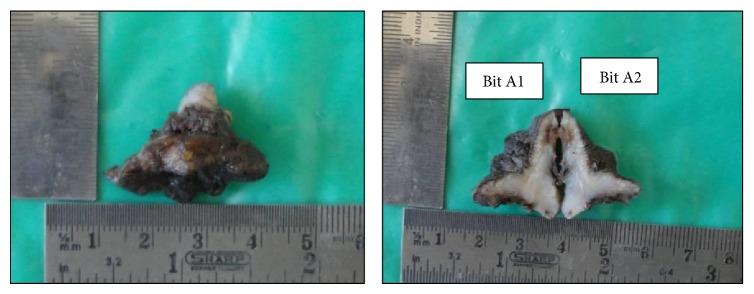
Gross specimen photograph of the excisional biopsy.

**Figure 5:**
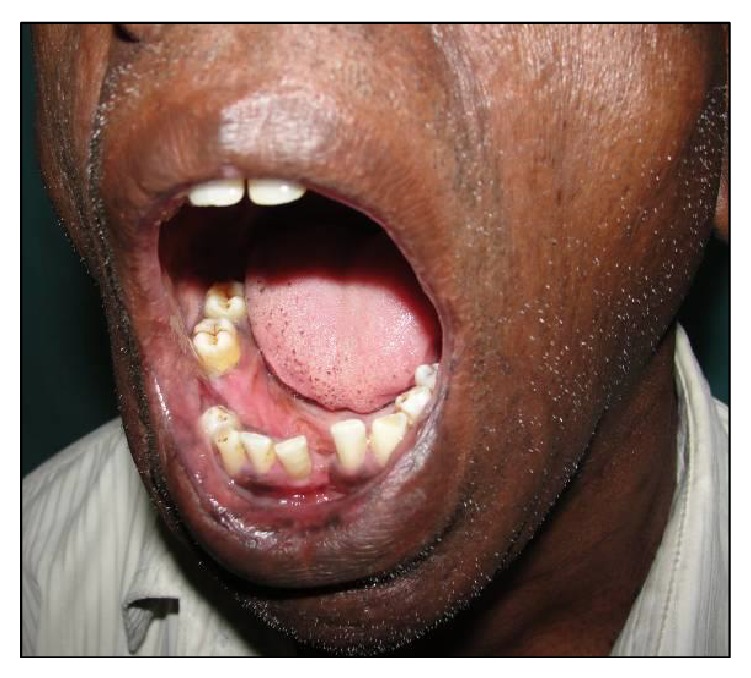
Clinical photograph of post-op intraoral choristoma.

**Figure 6:**
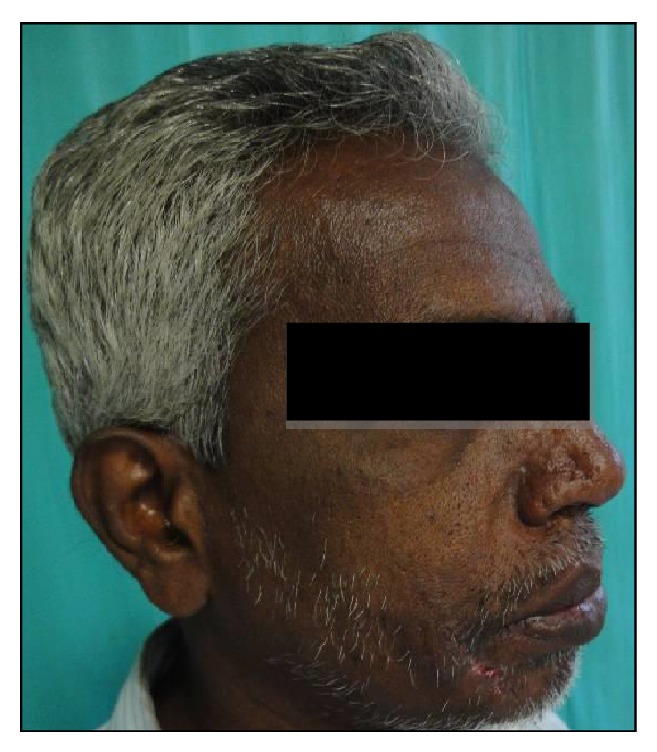
Clinical photograph of post-op extraoral choristoma.

**Figure 7:**
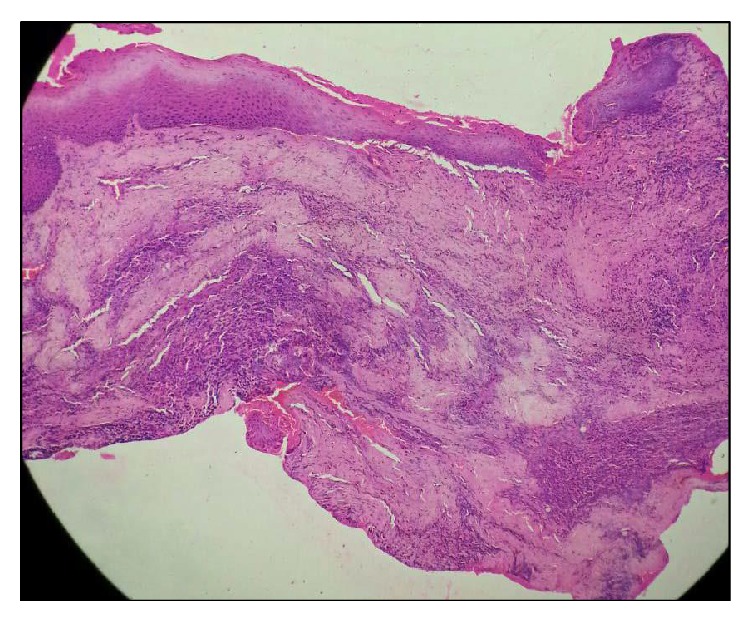
Section showing parakeratinized stratified squamous epithelium with cartilaginous islands and inflammatory cell infiltration in the connective tissue stroma (H&E 20x).

**Figure 8:**
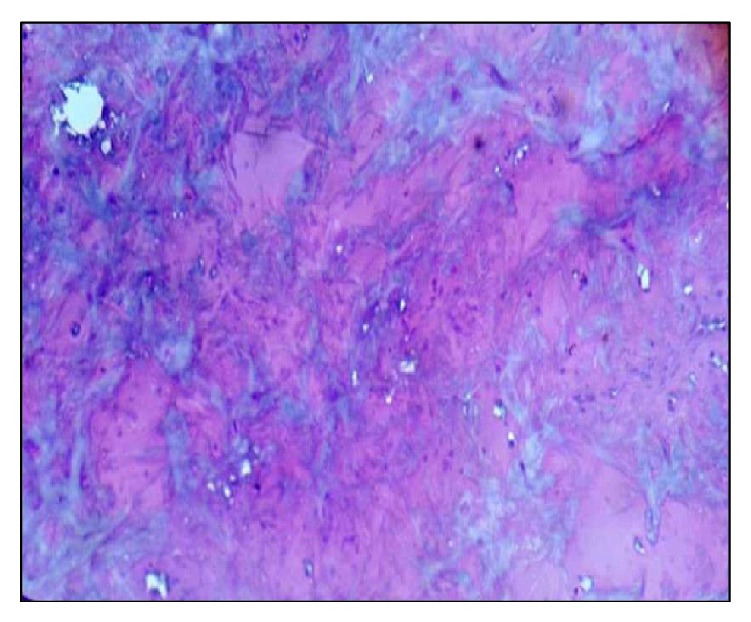
Section showing islands of basophilic mature cartilaginous tissue (H&E 40x).

A 54-year-old male reported to the Department of Oral and Maxillofacial Surgery (OMFS) with swelling of gums on right lower posterior region of mouth for 5 years (Figure 1). The swelling gradually increased in size associated with throbbing type of pain. A single sinus tract was present on the right side of the face (Figure 2). Incisional biopsy was carried out and specimen was sent to the Department of Oral Pathology and Microbiology with a provisional diagnosis of epulis. The gross specimen measured around 1.7 cm × 0.5 cm × 0.3 cm in dimension, which was soft to firm in consistency, white in color, and cylindrical in shape, with a lobulated surface (Figure 3). The histopathological examination revealed peripheral parakeratinized stratified squamous epithelium. The underlying connective tissue stroma showed well-circumscribed mass of basophilic mature cartilage. Excisional biopsy was performed under general anaesthesia. The grossed specimen measured around 4 cm × 1.5 cm × 0.6 cm in dimension and was greyish black intermixed with creamish white in color, firm to hard in consistency, and roughly triangular in shape, with irregular surface which was further grossed into two equal halves (Figure 4). Histopathological examination showed islands of basophilic mature cartilage in a dense fibrous connective tissue stroma along with focal collection of chronic inflammatory cells, chiefly plasma cells and lymphocytes. Peripheral stratified squamous epithelium was noted (Figures 7 and 8). A final diagnosis of cartilaginous choristoma was given. There was no evidence of recurrence in a 3 year follow-up (Figures 5 and 6).

## 3. Discussion

Choristomas are dysmorphic proliferation of cells that are not resident to the organ in which they arise and, like hamartomas, they gain a certain size and then cease to grow. The term choristoma was first introduced by Krolls et al. [2]. Some researchers refer to choristoma as a tumor-like growth which has urbanized from groups of primitive cells located at a site far-off from the original tissue or organ [3]. Heterotopic gastrointestinal cyst may be initiated in the tongue or floor of the mouth of infants which contains gastrointestinal glandular structures and is considered as choristoma. The unusual finding of bone or cartilage in the tongue and the occasional growth of thyroid tissue in the posterior tongue are considered as choristoma. The reasonably frequent findings of Fordyce granules essentially represent choristomas that start as nonfunctional sebaceous glands that originate in the submucosal region. The uncommon enteric replication cyst in the floor of mouth and osteoma in the tongue are also patterns of choristomas. Salivary gland tissue within lymph nodes may also be considered as choristomas [4]. There are various discussions about whether choristomas are developmental, neoplastic, or reparative in character [5]. More than a few contributors support embryonic rests as a cause of gingival choristomas. It is also understood that pluripotent mesenchymal cells differentiate into osteocytes or chondrocytes [3].

Cartilaginous choristoma was initially explained by Berry in 1890 [6]. The occurrence of this lesion varied notably ranging from 10 to 80 years. It occurs more commonly in females. But, in our case report, the patient was a 54-year-old male. Oral cavity is the most frequent site of predilection in the head and neck area for cartilaginous choristoma [7].

Cartilaginous choristoma is apparently seen as a trouble-free, firm swelling [8]. This was seen in our case also where the patient visited the dentist 5 years from the period of its occurrence. Tongue is the most frequent location of occurrence in the oral cavity [8]. But, in our case, it involved the lower gingiva which is one of the rarest sites of occurrence of cartilaginous choristoma. Perrotti et al. reported a case of cartilaginous choristoma of the gingiva [9].

Cartilaginous proliferations of orofacial extraskeletal soft tissues apparently reflect the multipotential nature of primitive mesenchymal cells of that part of the body which may be stimulated to grow by trauma, irritation, or inflammation [10]. In this case, the swelling would have been due to proliferation of orofacial extraskeletal soft tissues which may have been stimulated by local factors such as calculus or poor oral hygiene of the patient.

Cartilaginous choristoma should be differentiated from cartilaginous metaplasia, which generally occurs in the soft tissue underneath ill-fitting dentures. On the contrary, our patient was a dentate person. Histopathologically, cartilaginous metaplasia is characterised by scattered deposits of cartilaginous cells and calcium that are arranged in a variety of phases of maturation in solitary or clustered cartilaginous foci [11].

Histopathological examination of our case showed islands of basophilic mature cartilage in a dense fibrous connective tissue stroma with focal collection of chronic inflammatory cells, chiefly plasma cells and lymphocytes. The overlying peripheral epithelium was noted as parakeratinized stratified squamous epithelium.

## 4. Conclusion

This case report highlights an occurrence of cartilaginous choristoma in a rare site—the gingiva in a male patient. This paper lays stress on the fact that cartilaginous choristoma is completely hamartomatous in nature and should be treated appropriately.

Early and precise diagnosis can be made with a great understanding of this entity. The intraoral choristoma is mainly treated with surgical excision. Recurrences have not been reported in most of the cases with follow-up [12].

## References

[1] Kumar V., Abbas A. K., Fausto N., Aster J. (2010). *Robbins and Cotran Pathologic Basis of Disease*.

[2] Krolls S. O., Jacoway J. R., Alexander W. N. (1971). Osseous choristomas (osteomas) of intraoral soft tissues. *Oral Surgery, Oral Medicine, Oral Pathology*.

[3] Chou L., Hansen L. S., Daniels T. E. (1991). Choristomas of the oral cavity: a review. *Oral Surgery Oral Medicine and Oral Pathology*.

[4] Marx R. E., Stern D. (2003). *Oral and Maxillofacial Pathology: A Rationale for Treatment*.

[5] Psimopoulou M., Antoniades K. (1998). Submental osseous choristoma: a case report. *Journal of Oral and Maxillofacial Surgery*.

[6] Berry J. (1890). Fibro-chondroma of tongue. *Transactions of the Pathological Society of London*.

[7] Naresh Bharti J., Ghosh N., Arora P., Goyal V. (2013). Chondroid choriostoma of palatine tonsil—a rare entity. *Journal of Clinical and Diagnostic Research*.

[8] Kannar V., Prabhakar K., Shalini S. S. (2013). Cartilaginous choristoma of tonsil: a hidden clinical entity. *Journal of Oral and Maxillofacial Pathology*.

[9] Perrotti V., Fioroni M., Rubini C., Piattelli A. (2005). Cartilaginous choristoma of the gingiva. *Oral Oncology Extra*.

[10] Kapoor N., Bhalla J., Bharadwaj V. K., Kotgirwar B. K. (2003). Cartilagenous choristoma of palatine tonsil—a case report. *Indian Journal of Pathology and Microbiology*.

[11] Cutright D. E. (1972). Osseous and chondromatous metaplasia caused by dentures. *Oral Surgery, Oral Medicine, Oral Pathology*.

[12] Goswamy M., Tabasum S., Kudva P., Gupta S. (2012). Osseous choristoma of the periodontium. *Journal of Indian Society of Periodontology*.

